# Exploring Communities: Use of Windshield Surveys in Pediatric Residency Education

**DOI:** 10.1177/00099228261463366

**Published:** 2026-07-07

**Authors:** Ana C. Monterrey, Emma A. Omoruyi, Nancy R. Kelly, Shilpa Pai, Christin Traba, Aditi Gupta

**Affiliations:** 1 Baylor College of Medicine, Houston, TX, USA; 2 McGovern Medical School UT Health Science Center at Houston, USA; 3 University of Texas Southwestern Medical Center, Dallas, USA; 4 Rutgers-Robert Wood Johnson Medical School, New Brunswick, NJ, USA; 5 Rutgers New Jersey Medical School, Newark, NJ, USA

**Keywords:** windshield survey, medical education, social determinants of health, community health, GME

## Abstract

Windshield surveys (WS) have been used to teach about social determinants of health (SDOH) through direct observation of communities. Limited information exists about how pediatric residency programs design and implement WS; therefore, the authors created a survey to understand their use. Twenty-one percent (61/290) of pediatric residency programs responded and 42 report using WS. Most require completion during first year of training (19/33). Learning objectives for the WS included themes on barriers to health, understanding resources and community characteristics. Resident assessment involved discussion with faculty (15/33), reflective writing (3/33), faculty review of WS (1/33), or a combination of these (8/33). To evaluate the curriculum, discussion (17/33), formal evaluations (7/33), or both (3/33) were used. Experiential activities like WS can teach about communities and SDOH, however, no standardization of methods or evaluation exist. Further research is needed on effective learner assessment and curriculum evaluation methods for residency programs wanting to implement WS.

## Introduction

Understanding social determinants of health (SDOH) is important for practicing pediatricians to better address health disparities facing the communities they serve.^1,2^ In fact, the US Accreditation Council for Graduate Medical Education (ACGME) Pediatric Program Requirements explicitly state all pediatric trainees need to receive training on SDOH.^3^ While some aspects of SDOH can be taught in a traditional classroom setting, windshield surveys or walking tours (referred to collectively here as WS) can provide robust learning through direct observation of the communities served by trainees. Windshield surveys or walking tours are a visual assessment from a car or on foot to develop an overview of a community or neighborhood’s characteristics traditionally used in the public health sector to conduct community assessments.^4,5^ Importantly, they have also been implemented into the curriculum of academic medical institutions,^6-10^ health professions training programs (eg, nursing and public health),^11-18^ and graduate medical education (GME) programs in various specialties^19-26^ as an experiential learning activity to teach about community health and SDOH. Windshield surveys or walking tours and community tours (adapted WS with community member input or guidance) have been shown to increase learner knowledge about community resources,^22,27^ increase awareness of community needs and strengths,^19,22,25,27,28^ and allow learners to reflect on how these relate to health inequities^21,28^ impacting their patients. Therefore, WS are an ideal tool to teach about SDOH in pediatric residency programs.

However, limited programmatic information is available about how pediatric residency programs design and implement WS. Typically, WS are mentioned only briefly as a component of larger curricula.^6-10,12-15,23-26,29^ Studies that elaborate further, still provide incomplete information regarding design, implementation, evaluation and assessment methods of learners.^16,17,21^ Resources are available with a basic framework and some guidance for conducting WS including the CDC’s *Heart-Healthy & Stroke Free: A Social Environment Handbook*,^5^ Anderson and MacFarlane’s book on public health nursing – *Community as Partner: Theory and Practice in Nursing*^11^ and the University of Kansas’ *Community Toolbox*.^30^ However, most of this content is focused on public health interventions and thus is not targeted at GME. The recently released *Community Asset Mapping Tool Guide for Faculty* from the American Academy of Pediatrics (AAP)^31^ and some studies focusing on GME programs^20-22,25,26^ still lack sufficient depth to support residency programs seeking guidance on how to develop curricula with WS for their own trainees. Furthermore, these resources do not detail other aspects of implementation such as timing within residency training, learner assessment or evaluation methods of this teaching tool. Given such limited programmatic information on WS in GME, our working group from the AAP Community Pediatric Training Initiative (CPTI) conducted a landscape analysis to compare implementation of WS as well as resident assessment and program evaluation methods in pediatric and medicine-pediatric residency programs in the United States.

## Methods

### Survey Design and Questions

A group of pediatric faculty educators were identified through the AAP CPTI to create a survey mapping the usage of WS in pediatric and medicine-pediatric combined residency training programs in the United States. Using an iterative process, the group developed a structured questionnaire or survey based on the current literature and their expertise using WS in GME. Faculty piloted the survey to ensure appropriate data collection prior to finalizing. Multiple-choice questions explored development of WS including implementation practices (when, where, and how WS are conducted) and related assignments. Critical elements of educational interventions include learning objectives, and methods for assessment and evaluation.^32^ Therefore, programs were asked to attach a copy of their educational objectives and share what techniques they used to assess trainees’ learning and to evaluate the curriculum. Respondents were given the option to attach a copy of their full learning objectives, but no specific information was asked about effectiveness of the curriculum or follow up with learners. They were also asked general characteristics about the residency program including name, location, size and whether medicine-pediatric residents are included. In addition, respondents were asked to indicate their role in the residency program and familiarity with the program’s WS. None of the survey’s individual questions required a mandatory response and survey respondents could opt out of all or parts of the survey.

### Data Collection

The survey targeted faculty knowledgeable about community health training at their residency program. To recruit responses, an electronic link was sent twice via email (August-November 2021) to members of 3 pediatric medical organization groups focusing on resident education in community health and advocacy including AAP CPTI, Academic Pediatric Association Advocacy Training Special Interest Group, and the Association of Pediatric Program Directors Advocacy Training. Respondents were asked to complete the survey electronically via RedCap. Demographics for both responding and non-responding residency programs (name, city, and state) were collected from the Association of American Medical Colleges (AAMC) Electronic Residency Application Service (ERAS) Directory for US Pediatrics and Internal Medicine/Pediatrics Residency Programs.^33^ The AAP and the lead author’s institution granted institutional review board’s exemption and approval, respectively.

### Data Analysis

Survey responses were reviewed and those completed by non-faculty members who did not lead or have knowledge of the curriculum were excluded. Duplicate responses from the same residency program were also excluded. Data analysis included descriptive statistics with calculation of frequencies for quantitative results. The program demographics of survey responders and non-responders were compared using Fisher’s exact and chi-square tests. Thematic analysis^34^ of the WS learning objectives was conducted by 2 authors (ACM and AG) who independently reviewed and manually coded the objectives for recurring themes using an iterative and inductive coding framework. The authors then reviewed the themes together. Via discussion that took into account accuracy and consistency of codes, they developed a consensus of recurring themes. The frequency of each theme within the learning objectives was counted.

## Results

We received responses from 61/290 (21% response rate) pediatric programs in the U.S. Importantly, those that responded were reflective of all US-based pediatric programs with no difference found between responders and non-responders in terms of program location (Fisher’s exact test; *P* = .08) or program type (chi-square test; *P* = .34) (Table 1). There were 42/61 (68.5%) programs that indicated they include WS in their training. Since no individual survey questions (items) were required, this resulted in variable denominators across items for analysis. However, 33 of the programs utilizing WS answered at least 90% of the questions in the survey, providing the largest sample to date of programs that include WS in their curricula.

**Table 1.** US Pediatric Residency Programs.

	Survey nonresponders (N = 230)		Survey respondents (N = 61)		*P* value
Location					Fischer’s exact test
South	77	33.5%	27	44.3%	*P* = .08
NE	65	28.3%	12	19.7%	
Midwest	58	25.2%	10	16.4%	
West	25	10.9%	12	19.7%	
Other (Puerto Rico)	5	2.2%	0	0%	
Program type					χ^2^
Pediatrics only	101	43.9%	31	50.8%	*P* = .34
Includes medicine-pediatrics	129	56.1%	30	49.2%	

### Methods for Implementing WS Varied by Program

A primary aim of this study was to understand how GME programs implement WS into their training. Key aspects of implementation were assessed including: when, where, and how; related assignments and learning objectives.

*When*: Most programs complete their WS in PGY1 (19/33, 57.6%), 4/33 (12.1%) during PGY2, none during PGY3 only, and 10/33 (30.3%) complete the WS more than once during training (Figure 1A). Most programs reported completing the WS as part of a required community and advocacy training block (28/34, 82.4%), 5/34 (14.7%) during residency orientation, and 1/34 (2.9%) program includes it as part of an elective rotation.

**Figure 1.**
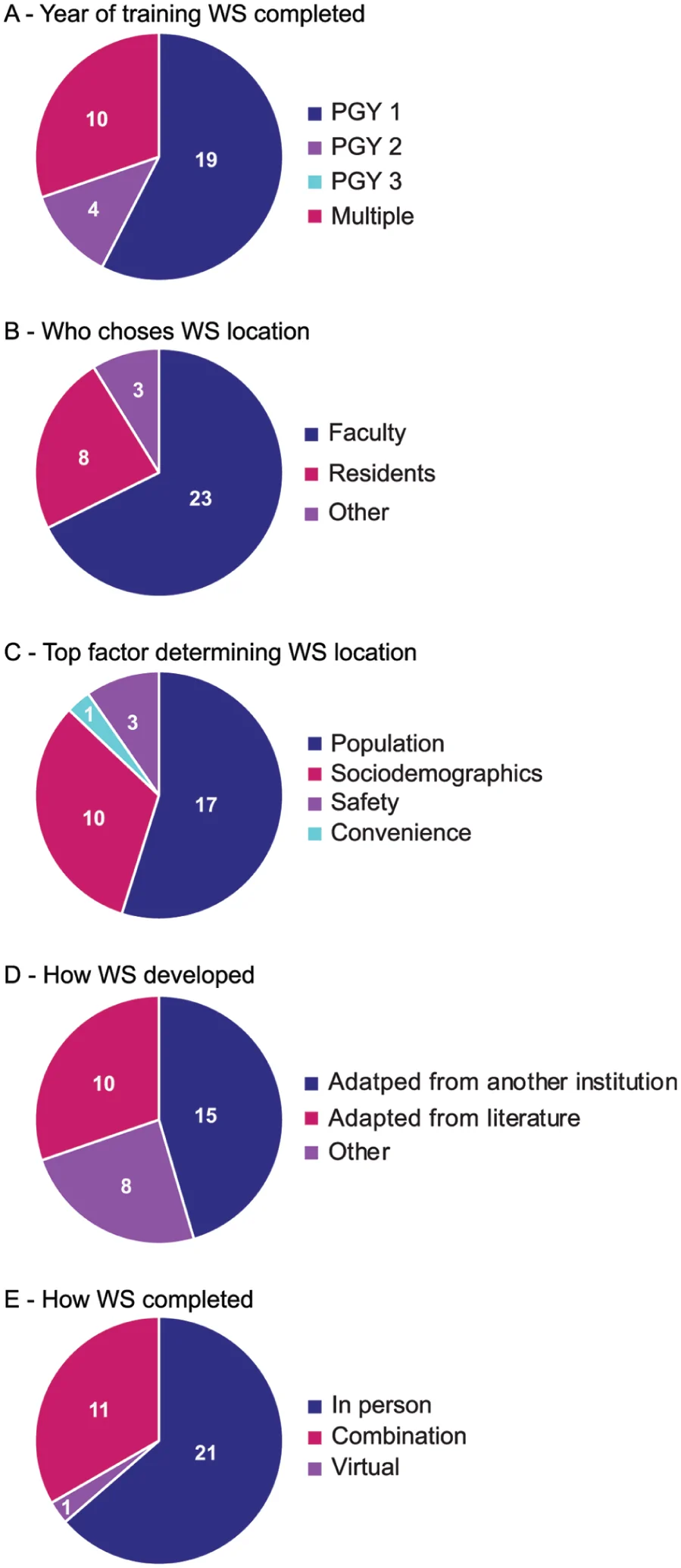
Implementation features for WS.

*Where*: Regarding who chose the location, faculty most commonly decided (23/34, 67.7%), while some programs allowed residents to choose (8/34, 23.5%), and the rest used a combination of these (3/34, 8.8%) (Figure 1B). When asked to rank the factors that were most important, respondents most commonly cited the hospital or clinic’s surrounding population as the top factor determining the neighborhood where WS were conducted (17/31, 54.8%). Sociodemographics of the neighborhood were the next top ranked (10/31, 32.3%). Factors like safety (3/31, 9.7%) or convenience (1/31, 3.2%) were less important (Figure 1C).

These data suggest that most programs who use WS require this activity early in their training and that typically faculty choose where the WS will be conducted to expose residents to the neighborhoods where their patients live and the demographic of patients they will be caring for.

*How*: Regarding programmatic design, nearly half of programs (15/33, 45.5%) adapted their WS from another institution, 10/33 (30.3%) used the literature and 8/33 (24.2%) used other methods such as faculty expertise and experience working with the community (Figure 1D). Most also have residents follow a standardized template (22/34, 64.7%). Many programs complete the WS in person either via car/bus or walking/biking (21/33, 63.6%), while the rest complete it virtually (1/33, 3%) or use a combination of both methods (11/33, 33.4%) (Figure 1E). Respondents reported that residents complete the WS individually (10/33, 30.3%), in groups (13/33, 39.4%) or using a combination of both methods (10/33, 30.3%).

*Related assignments*: Only 11/34 (32.4%) programs required any preparatory work in advance of completing the WS and only 13/33 (39.4%) asked residents to compare and contrast 2 or more neighborhoods. Ten programs of 33 (30.3%) noted that the WS informed a larger project or intervention such as an advocacy or community project.

In summary, while there is some variety in how WS are implemented, most programs standardize the activity for all residents using a template tool completed early in training so residents can begin to observe the communities they will work with firsthand.

#### Five Key Themes Essential to WS Program Learning Objectives

More than half of programs (21/32, 65.6%) had learning objectives for their WS and 17 programs shared their specific curriculum objectives. On review of these objectives, 5 key themes were identified (Table 2). The most common theme was for residents to *gain an understanding of the challenges and barriers to health experienced by patients in the community* (N = 11). The next most common theme was for residents to *identify resources and assets in the communities they work with* (N = 10). Having residents *develop an awareness of the social drivers that impact the health of their patients* was another common theme among the learning objectives (N = 8) in addition to *providing residents with a basic understanding of the characteristics of the community* (N = 6). Finally, the objectives also focused on *recognizing the importance of diversity among communities and the role bias plays when providing care* (N = 5).

**Table 2.** Learning Objectives Recurring Themes.

**Challenges or barriers (N = 11)**
“Identify, analyze, and describe health disparities, as well as organizational assets and barriers to delivering culturally effective services.”
“Explore community barriers to health.”
“[Discuss] needs and challenges that impact the health and well-being of children and families.”
“Explain challenges patients face in accesses services.”
**Resources and assets (N = 10)**
“Identify local community resources and the services they offer patients.” “Identify community assets and resources.”
“Recognize various community organizations and resources which are valuable to our patients.” “Increase awareness of basic community resources available to our patients in their neighborhood.”
**Recognizing social drivers impacting community (N = 8)**
“Incorporate knowledge of structural determinants of health into the counseling.”
“Recognize the influence of physical space and the built environment on health.”
“Reflect upon how race, poverty, schools, health care access, food access, and the built environment intersect to impact the health of individuals and communities . . .”
**Basic understanding/characteristics of community (N=6)**
“Describe the socio-demographics that relate to the health of kids and families in the community.”
“Identify unique characteristics of the neighborhoods . . .”
“Better understand the environment you are working in.”
**Importance of diversity and role of bias (N = 5)**
“Integrate into clinical encounters an understanding of diversity [. . .] by recognizing and respecting families’ cultural backgrounds.”
“Identify and manage cultural attributes, stereotypes, and biases you bring to clinical encounters.”
“Reflect on personal values, beliefs, and assumptions about the ways that health of individuals and communities is impacted by the social, cultural, economic, environmental and structural factors.”

### Assessing Resident Learning From WS Is Challenging

There were 6/33 (18.2%) programs that reported they did not include any method of resident assessment and only required completion of the activity. The rest of the programs reported varied methods, including 15/33 (45.5%) programs using a discussion session led by faculty, 3/33 (9.1%) using reflective writing, and 1/33 (3%) having faculty review the WS and give feedback. A combination of all these methods was used by 8/33 (24.2%) programs (Figure 2A). A final key element of curriculum development is program evaluation. When asking programs how they evaluate the curriculum and obtain feedback from residents, 6/33 (18.2%) programs reported not obtaining any feedback on the activity. Otherwise, 17/33 (51.5%) programs reported using in person/virtual discussions with residents, 7/21 (21.2%) obtain formal evaluation feedback and 3/33 (9.1%) use a combination of both methods (Figure 2B). Therefore, we conclude that assessment of learners and evaluation of the curriculum is more variable among programs.

**Figure 2.**
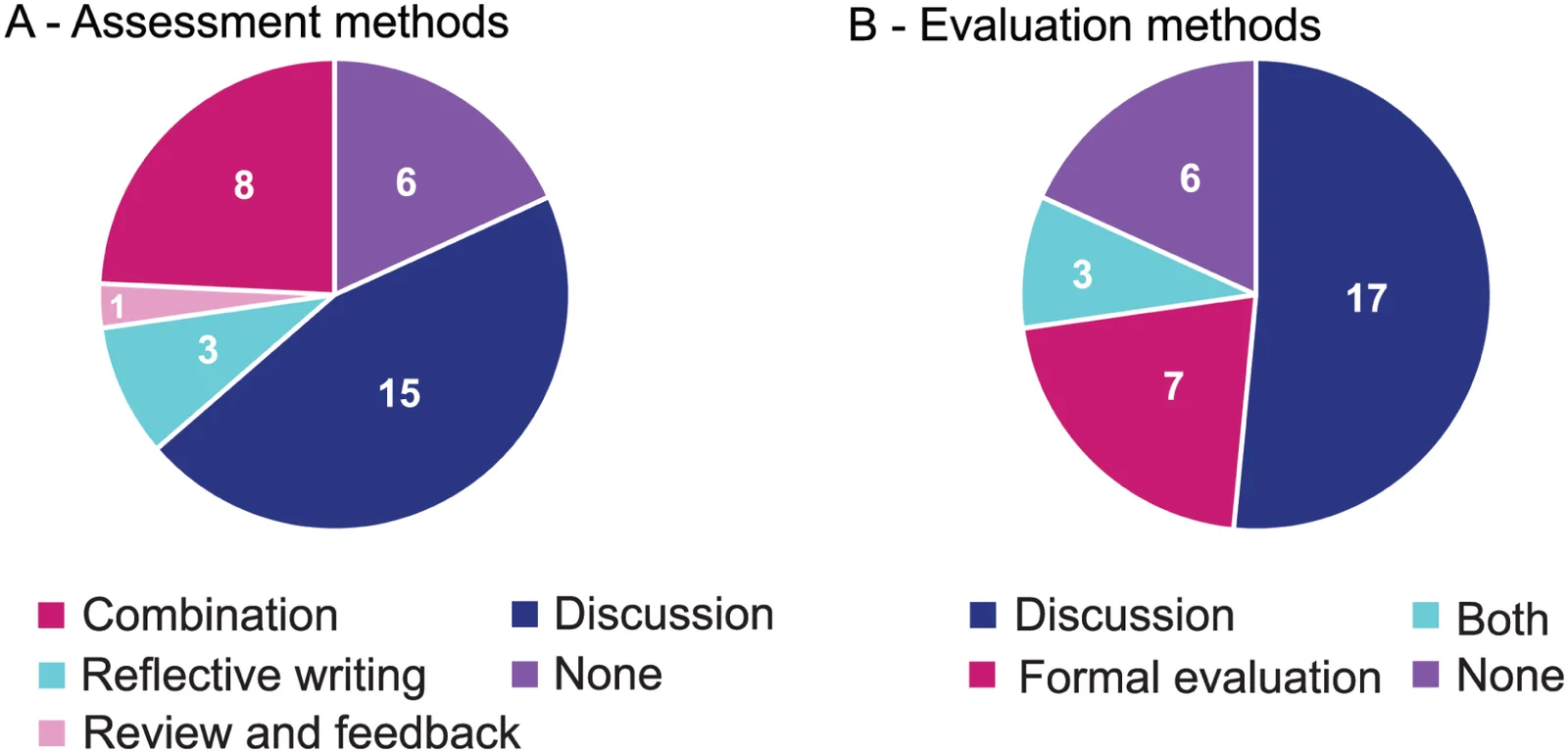
Resident assessment and program evaluation methods.

## Discussion

In this study, we surveyed faculty members of pediatric medical organization groups focusing on resident education in community health and advocacy to understand how WS are implemented in GME training. We found that methods varied, but most programs utilized WS early in training using a standardized template in an area chosen by faculty to help trainees learn about the important aspects of the community such as barriers and assets to care and available resources. Program evaluation was more varied and is likely an important area for future study.

### Resident Assessment and Program Evaluation of WS Is Difficult

Evaluating SDOH curricula like WS, including assessing resident learning, can be difficult. While SDOH education in GME is now recognized as an important aspect of the training,^35^ review articles on SDOH education in GME, undergraduate medical education (UGME) and allied health programs note gaps still exist among these activities, especially related to learner assessment methods and long-term evaluation.^36-40^ Our study found that despite having many similar learning objectives, how programs assessed resident learning and programmatic evaluation to ensure curriculum effectiveness varied considerably. Some programs did not assess learners or obtain curriculum evaluation data at all. Similarly, a scoping review by Nour et al,^36^ in 2024 only included 24 articles across all GME specialties that met inclusion criteria to have a structured SDOH curricula with accompanying programmatic evaluation. Even across these studies, evaluation methods were heterogeneous, including pre/post surveys, group and individual discussions, written reflections, and focus groups or a combination of these to assess learner knowledge and attitudes on SDOH. In addition, only 2 of the 24 articles in this scoping review assessed resident performance (self-reported) as part of their evaluation measures and only 1 incorporated patient views on care provided after participation in SDOH curricula. This lack of objective assessment of learner skills or change in behavior is common in the literature describing SDOH curricula in both GME and UGME.^37,38,40,41^ Importantly, SDOH education has been criticized for failing to assess its impact on patient health outcomes and how it translates to value for communities.^1,42,43^ Our data reflect this trend, as the learning objectives for WS were mostly focused on resident knowledge and understanding, and not on patient care skills. Therefore, this is a targetable area for improvement and important to ensure these curricula are meeting their intended goals. Simulation or objective structured clinical examinations or assessments have been identified as a way to assess impact on learner skills;^40,41,43^ however, this is not widely used as it likely challenging and costly for GME programs to implement. Direct observation of skills in the care of patients would require significant time from faculty as well as other approvals and ethical considerations due to involving patients. Future research should focus on how to effectively evaluate SDOH curricula such as WS to support GME programs that wish to implement them and objectively show impact on resident skills, while still considering limited time and resources.

### WS Can Help GME Programs Meet ACGME Requirements Related to Collaborating With Communities and Population Health

Pediatric ACGME requirements and pediatric Entrustable Professional Activities (everyday pediatrician activities needed to meet the needs of patients) include competencies related to understanding the context of the community where patient care occurs and collaborating with these communities to improve health,^44,45^ but these objectives can be challenging to achieve. The majority of programs encouraged residents to directly observe the neighborhoods where their patients live, with the goal of understanding community characteristics and the social drivers impacting them, suggesting that activities like WS can meet this requirement. The 2 most common learning objective themes were to have residents reflect on barriers to health care and resources that may support health. All of this is vital for practicing physicians to begin understanding the underlying causes of health disparities and ways they may be able to combat these and improve public health.^1,2,43^ Pediatric GME programs also require scholarship which can be fulfilled by projects focusing on population health topics.^44^ Activities like WS can help residents reflect on opportunities to become involved in community service during training or may lay the foundation as they develop scholarly projects collaborating with the community to promote health.^1^ Indeed, 10 respondents in this study reported using WS this way to inform a larger project residents complete. Thus, future research evaluating how to leverage WS in developing community and advocacy projects will be beneficial. Windshield surveys are also a simple tool residents can use as they begin to integrate themselves into the communities they will work with and as practicing pediatricians can be used to better familiarize themselves with the patient populations they will care for. Therefore, implementing activities like WS into pediatric residency curricula is valuable for meeting program requirements and fostering skills related to working with communities.

## Limitations

There were several limitations to our study including a potential for selection and response bias and a low response rate from pediatric and medicine-pediatric combined residency programs. Due to the nature of recruitment via professional organization mailing lists, which did not involve reaching out to each individual program director, it is possible not every program had the opportunity to reply. However, recruitment through the Association of Pediatric Program Directors was specifically included since it represents the primary national professional organization for pediatric residency leadership. Membership encompasses nearly all accredited pediatric training programs, thereby maximizing broad dissemination and national reach of our survey. However, given survey participation was voluntary, respondents may have included programs with a stronger interest in community health and advocacy education. Furthermore, some institutions with both categorical pediatric and medicine-pediatric combined programs may include WS as part of a curriculum both groups of residents participate in together. Therefore, some of our survey responses may reflect more than one residency program, further impacting our response rate. While this low response rate may limit generalizability of the study, the responses we did receive were reflective of both pediatric and medicine-pediatric residency programs distributed across the 4 major US census regions.

Finally, while our survey asked about learner assessment and programmatic evaluation methods, we did not ask respondents for outcome measures about their curricula. We are unable to comment on how effective these WS are at meeting their intended educational learning objectives and whether any programmatic follow up occurred with learners.

## Conclusion

Windshield surveys are an experiential learning activity used to teach residents about SDOH in the communities they will serve during their training. This landscape analysis on the use of WS in pediatric residency education found that while some elements of implementation are similar, methods for learner assessment and curricular evaluation are more varied. In addition, further research is needed to evaluate these curricula and objectively show their impact. The goal of teaching about SDOH is for residents to incorporate an understanding of social drivers into the care they provide patients, but behavioral changes represent a higher level of learning which can be more challenging to assess in the long term. Developing clear learning objectives and outcomes will be important for programs to begin addressing this issue. Further research needs to focus on effective learner assessment and curriculum evaluation methods for residency programs wanting to implement or enhance their WS. Finally, another area of development includes understanding the role WS can play in developing scholarly projects promoting population health. Windshield surveys could be an important first step for trainees developing community interventions or for participating in advocacy activities focused on reducing health disparities, as they can provide some perspective and understanding of the potential health needs of their patient populations. They can also provide residents with a simple tool to use as future pediatricians and advocates to get to know and collaborate with the communities they serve.

## Author Contributions

AM, EO, and AG: Contributed to material preparation, data collection, and analysis.

AM: Contributed to the first draft of the manuscript.

All authors contributed to the study conception and design, commented on previous versions of the manuscript, and read and approved the final manuscript.

## Supplemental Material

sj-pdf-1-cpj-10.1177_00099228261463366 – Supplemental material for Exploring Communities: Use of Windshield Surveys in Pediatric Residency Education Supplemental material, sj-pdf-1-cpj-10.1177_00099228261463366 for Exploring Communities: Use of Windshield Surveys in Pediatric Residency Education by Ana C. Monterrey, Emma A. Omoruyi, Nancy R. Kelly, Shilpa Pai, Christin Traba and Aditi Gupta in Clinical Pediatrics

## Data Availability

The datasets generated during and/or analyzed during the current study are not publicly available as participants of this study did not give written consent for their data to be shared publicly.
