# Supplementary material for: Exploring Communities: Use of Windshield Surveys in Pediatric Residency Education
Source: Clin Pediatr (Phila). 2026 Jul 7;65(11):1290–8. doi: 10.1177/00099228261463366 (PMC13672880; doi:10.1177/00099228261463366)
Supplement: Supplementary file 1 [file sj-pdf-1-cpj-10.1177_00099228261463366.pdf]

Residency Program Name:

____

City and State in which residency program is located:

____

Approximate size of residency program (number of residents/year):

____

Does your program include Med/Peds residents?

◯ No
◯ Yes

What is your role within the residency program?

◯ Program Director
◯ Associate Program Director
◯ Advocacy or Community Health director
◯ Other

Please describe:

____

Do your residency program have a community health or advocacy curriculum?

◯ No
◯ Yes

Does your residency program have windshield survey or walking survey activity to assess neighborhoods and/or the built environment?

◯ No
◯ Yes

We would like to learn more about how your program has implemented the WS activity.

Are you in charge of or the person most knowledgeable about the WS activity?

◯ No (Please consider not completing the survey further and forwarding to the appropriate person at your program.)
◯ Yes

Do you have learning objectives specifically for your program's WS activity?

◯ No
◯ Yes

What are the objectives for the WS activity? Please type here:

As part of the WS activity, do your residents complete or follow a tool/template?

◯ No
◯ Yes

If you are willing, can you please attach a copy of your WS tool/template here?

How did you develop the WS activity tool/template?

◯ Adapted from the literature
◯ Adapted from another institution or colleague
◯ Other

Please describe:

____

Do your residents complete any pre-work/preparation prior to the WS activity?

◯ No
◯ Yes

What kind of preparation is done? (check all that apply)

☐ Introduction discussion/lecture
☐ Review of community health metrics or statistics (e.g. using KidsCount Data or other database)
☐ Tour of the community (including during intern orientation or other rotations)
☐ Other

Please describe:

____

During what year of residency training is the WS activity done? (check all that apply)

☐ PGY1
☐ PGY2
☐ PGY3+
☐ Multiple years (ie. It is completed more than once during residency)

Please describe:

____

During which residency rotation is WS activity done?

◯ Required community or advocacy rotation
◯ Elective
◯ Other

Please describe:

____

Who decides where your residents will conduct the WS activity?

◯ Faculty
◯ Residents
◯ Other

Please describe:

____

What factors determine the choice of neighborhood where WS activity is conducted? (Rank those that apply, 1=Most important, 4=Least important)

	1	2	3	4
Hospital/Clinic/Continuity population	◯	◯	◯	◯
Sociodemographics of the neighborhood (including race/ethnicity, economic factors)	◯	◯	◯	◯
Convenience	◯	◯	◯	◯
Safety	◯	◯	◯	◯

Do your residents compare and contrast 2 or more neighborhoods for the WS activity?

◯ No
◯ Yes

How do your residents complete the WS activity?

◯ Individually
◯ In groups
◯ Both

What method is used to conduct the WS activity? (check all that apply)

☐ Virtually (e.g. using googlemaps)
☐ In person driving through the community/neighborhood
☐ In person walking/biking through the community/neighborhood
☐ Other

Please describe:

____

Do your residents take any photos/videos during the WS activity?

◯ No
◯ Yes

What guidance if any, is given regarding photos?

____

What are the photos used for?

____

Are your residents using the WS activity to inform the development of a larger project or intervention? (e.g. home visitation curriculum, service/volunteer project or other community intervention/longitudinal project, etc)

◯ No
◯ Yes

Please describe:

____

How do you assess the WS activity? (Check all that apply)

☐ We do not assess (completion only)
☐ Discussion of the activity with faculty
☐ Faculty review completed tool/template and provide feedback
☐ Resident reflective writing assignment
☐ Other

Please describe:

____

How do you receive/obtain feedback from your residents on their participation in the WS activity?

◯ In person/virtual discussion
◯ Formal evaluation feedback
◯ No feedback obtained
◯ Other

Please describe:

____

Have you made any changes to the WS activity due to COVID-19?

◯ No
◯ Yes

Please describe:

____

Is there anything else you would like to share regarding your residency program's use of the WS activity?
